# PSMA radioligand uptake correlates with PSMA expression in high-grade glioma and brain metastasis: insights from a prospective PET-MRI guided multiregional biopsy study

**DOI:** 10.1007/s00259-025-07338-4

**Published:** 2025-05-26

**Authors:** Ilanah J. Pruis, Vera van Dis, Sybren L. N. Maas, Rutger K. Balvers, Thierry P. P. van den Bosch, Marcel Segbers, Sophie E. M. Veldhuijzen van Zanten

**Affiliations:** 1 Department of Radiology and Nuclear Medicine, University Medical Centre Rotterdam, Rotterdam, The Netherlands; 2 Brain Tumour Centre, Erasmus MC Cancer Institute, Rotterdam, The Netherlands; 3 Department of Pathology, Erasmus MC Cancer Institute, University Medical Centre Rotterdam, Rotterdam, The Netherlands; 4 Department of Pathology, Leiden University Medical Centre, Leiden, Netherlands; 5 Department of Neurosurgery, University Medical Centre Rotterdam, Rotterdam, The Netherlands; 6 Medical Delta, Delft, The Netherlands

**Keywords:** Prostate-specific membrane antigen, PET-MRI, Image-guided biopsy sampling, Immunohistochemistry, Malignant brain tumour

## Abstract

**Purpose:**

Prostate-specific membrane antigen (PSMA) is a potential target for radioligand therapy (RLT) in neuro-oncology. This study investigates the direct relation between [^68^Ga]Ga-PSMA-11 uptake on PET and PSMA expression in the tumour micro-environment of high-grade glioma (HGG) and brain metastasis (BM).

**Methods:**

Twelve patients with HGG (glioblastoma *n* = 6, oligodendroglioma *n* = 1), or BM (lung- *n* = 4, breast cancer *n* = 1), underwent PET-MRI after intravenous [^68^Ga]Ga-PSMA-11 injection (1.5 MBq/kg), followed by image-guided biopsy sampling during (re-)resection surgery. Multiple samples (median *n* = 3/patient, *n* = 23 HGG/*n* = 20 BM) from locations of low and high [^68^Ga]Ga-PSMA-11 uptake were analysed for PSMA expression in vasculature and non-vascular structures using morphology and immunohistochemistry.

**Results:**

All patients showed [^68^Ga]Ga-PSMA-11 uptake in tumour ( SUVmax median, range: 10.5, 4.7–19.8). Strong PSMA expression was found in tumour microvasculature (14/23, 61% in HGG, 13/20, 65% in BM). Tumour cell PSMA expression was found in a subset of HGG (10/23; of which strong in 8/10) and BM (3/20; none of which showed strong expression). Strong PSMA expression was also found on non-malignant glial cells in tumour. PSMA expression in healthy brain control samples was negligible. In HGG, a significant correlation existed between [^68^Ga]Ga-PSMA-11 uptake and PSMA expression in tumour microvasculature (r = 0.487, *P* < 0.01), but not tumour cells.

**Conclusion:**

PSMA expression in brain tumours is predominately vascular, which likely explains why microvascular (rather than tumour cell) PSMA expression correlates with [^68^Ga]Ga-PSMA-11 uptake in HGG. This neovascular expression is crucial information for future PSMA-based RLT studies, as alpha-emitters may not sufficiently target tumour DNA.

NCT05798273; date of registration: 1/9/2020.

**Supplementary Information:**

The online version contains supplementary material available at 10.1007/s00259-025-07338-4.

## Introduction

Novel therapeutic approaches that specifically target tumour cells or microenvironmental changes, and not pre-existent brain tissue, are highly desired to improve survival of patients with malignant brain tumours. In the field of neuro-oncology there is a growing interest for the concept of theranostics, which refers to the use of radionuclides linked to tumour-targeting ligands (radioligands), either for diagnostic imaging (by positron- or single-photon-emission tomography (PET/SPECT)) or for targeted radioligand therapy (RLT) [1]. Diagnostic radionuclides used most frequently in clinical practice are gamma particle-emitters (e.g., fluor-18 or gallium-68), which allow for target visualisation and uptake quantification. Therapeutic radionuclides mainly include beta-emitters (e.g., lutetium-177, holmium-166 and terbium-161), but also stronger alpha-emitters (actinium-225, lead-212) are entering the therapeutic arena to enable local DNA damage and cell death.

A recently discovered target with high potential for theranostic applications in glioma and brain metastases is prostate specific membrane antigen (PSMA); a transmembrane protein that is already successfully exploited for the treatment of prostate cancer patients [2]. To date, several immunohistochemistry studies found that PSMA is also highly expressed on the endothelial cells of neovasculature and, to a lesser extent, on tumour cells in high-grade glioma (HGG) and brain metastasis (BM) [3, 4]. Correlations were also found between the level of PSMA expression and tumour grade and survival [5, 6]. The first case series and clinical PET studies published to date [4, 7], confirmed moderate to high uptake of a variety of PSMA-based radioligands in patients with HGG and BM, both in primary and recurrent setting, while pseudoprogression/radiation necrosis and pre-existent brain tissue showed little to no uptake, indicating that PSMA is a potential target for RLT in neuro-oncology.

Essential prerequisites for successful RLT include specific binding of the radioligand to its biological target, and accumulation and retention in proximity to the tumour DNA, to ensure DNA damage and induction of tumour cell death [8]. The correlation between PSMA-based radioligand uptake on PET and PSMA expression in tissue has not been demonstrated before. One study found that high PSMA expression on endothelial cells in the tumour micro-environment, but not tumour cells, was correlated to high radioligand uptake on PET in eleven patients [9], however, in our own multicentre study we could not replicate these findings [10]. More extensive tumour tissue -sampling and -analysis can potentially better capture the heterogeneity of the tumour micro-environment to increase our knowledge on the PSMA expression patterns in relation to radioligand uptake dynamics on PET. Here, we performed a correlation study to investigate the relation between pre-operative uptake intensity of [^68^Ga]Ga-PSMA-11 on PET-MRI and PSMA expression in multiregional targeted tumour samples in patients with HGG or BM.

## Material and methods

### Patients

Approval for this study was obtained from the Medical Ethics Committee at Erasmus MC (NCT05798273). Patients were recruited during standard clinical visits to the department of neuro-oncology or neuro-surgery. Written informed consent was obtained from each patient in accordance with provisions of the declaration of Helsinki. Inclusion criteria were: radiologically-presumed and/or histologically-confirmed glioma showing enhancement on post-contrast MRI at time of primary diagnosis or progressive disease, or brain metastasis/-es, planned for (re-)resection, age ≥ 18 years old, good clinical condition (Karnofsky performance status ≥ 70), and ability and willingness to provide written informed consent. A detailed description of the enrolment (in- and exclusion) criteria and scanning protocol is described elsewhere [11].

A limited set of data from patient no. 1–5 has been described previously in a multicentre study, which extensively reports on the time activity curves and biodistribution of [^68^Ga]Ga-PSMA-11 [10]. For the present study, more extensive methods as described below, were used for and in-depth analysis of the PET-MRI data and samples.

As negative control, a slice of brain parenchyma taken from the frontal lobe from two control autopsy patients without a tumour were obtained from the biobank of Erasmus Medical Centre. This biobank tissue was stored with written informed consent from patients for scientific use of their data in accordance with provisions of the declaration of Helsinki.

### PET-MRI acquisition

Scans were acquired on a 3.0 T whole-body hybrid PET-MR system (Signa PET-MR, GE Healthcare, USA)) at 90 min (min; scan 1), 165 min (scan 2) and 240 min (scan 3) after intravenous injection of 1.5 MBq/kg (median, IQR 123, 99–134 MBq) of [^68^Ga]Ga-PSMA-11. At each timepoint, a single bed position (bp) PET scan of the brain was acquired for 15 min. The first scan was followed by a whole-body scan (from skull to vertex to thighs) of 5 bp (each 3 min). The image acquisition details are described in the Supplementary Material.

### Neuronavigation and surgical biopsy procedure

Prior to surgery, hybrid PET-MR images were reviewed by the neuroradiologist and nuclear medicine physician in collaboration with the neurosurgeon. Tumour regions with relatively low and high [^68^Ga]Ga-PSMA-11 uptake were identified by visual assessment and selected for biopsy.

For per-operative neuronavigation, hybrid PET and post-contrast T1w MR images from the third time-point (scan 3) were transferred into BrainLab © (BrainLab AG, Munich, Germany). In all patients, multiple image-guided samples were obtained, with the biopsy sites documented via intraoperative screenshots. Samples were formalin-fixed and paraffin embedded (FFPE) according to standard procedures for clinical samples.

### SUV uptake analysis

Spherical 1 cm^3^ volumes of interest (VOIs) were delineated for each biopsy location on all PET scans (i.e., timepoints 1–3), using BrainLab screenshot correlations to ensure spatial accuracy. This approach is consistent with similar studies investigating the spatial correlation between amino acid PET uptake and LAT1 expression [12, 13]. Within each VOI, the maximum uptake (SUVmax) was calculated using Hermes Hybrid Viewer V4.0.0. Based on our previous multicentre experience [10], the 240 min post-injection time point was selected for correlation with PSMA expression, as this scan consistently demonstrated stable and representative uptake across tumours. Additionally, SUVmean in the left ventricle was calculated using a spherical VOI with a diameter of 2 cm to serve as a reference value for blood pool activity. It should be emphasized that the whole-body scans were acquired between 105 and 130 min post-injection, and as such, blood pool values are expected to be slightly higher than would be expected at the 240 min post-injection time point.

### Immunohistochemistry analysis

FFPE blocks were sliced into 4-µm tissue sections. All tissue sections were separately (i) stained with Hematoxylin and Eosin (H&E) or (ii) immunostained with mouse-anti-PSMA (DAKO, M3620).

Stainings were evaluated independently by two neuropathologists (VD, SLNM) who were blinded for the results from the [^68^Ga]Ga-PSMA-11 uptake analysis on PET and for each other's scoring. First, H&E stainings were used to visually describe the morphology of each sample. For example, disorganized cells (clusters) were described as tumour cells, whereas well-organized cell clusters and scarred tissue (e.g. cell nuclei fragmentation, scattered fragments of brain parenchyma and gliosis) were described as pre-existent (i.e., normal) brain and necrotic tissue, respectively. The two pathologists independently estimated the percentage of each component (tumour, pre-existent brain tissue and necrosis) and scored the presence of microvessels within the tissue section area.

Subsequently, the H&E and PSMA stainings were used to measure the level of PSMA expression and visually assess the distribution across the sample components. The level of expression was scored according to a five-point scale, as used in our previous multicentre study [10], and similar PSMA immunohistochemistry studies [3]. This scale combines both intensity and extent of the staining to come to one PSMA expression score (0 = none, 1 = limited, 2 = moderate, 3 = strong, 4 = very strong). The pathologists’ pooled average of the two PSMA expression scores were used for the analyses. The inter-rater agreement between pathologists was calculated by grouping low (scores 0–2) versus high (scores 3–4) scores.

In each sample, the level of PSMA expression on microvasculature was scored separately from other non-vascular structures [6]. The level of PSMA expression on tumour cells was not separately scored as it can be challenging for the neuropathologist to distinguish tumour cells from normal glial cells and other TME components using H&E. When PSMA expression in individual tumour cells was identified this was recorded.

### Multiplex immunofluorescence staining of tumour-microenvironment

As an alternative to H&E, an assessment using multiplex immunofluorescence was performed in HGG patients 3, 4, and 5, which did allow for precise evaluation of the level of PSMA expression across the different tumour components. Following deparaffinization and heat-induced antigen retrieval with CC1 (#950–500, Ventana), multiplex immunofluorescence was performed on the Ventana Benchmark Discovery (Ventana Medical Systems Inc.). Multiplex stainings with rabbit-anti-PSMA (13 µg/mL, EP192; Cell Marque) were performed with the following markers: mouse-anti-nestin (1:25,600; NovusBio) for neuroepithelial stem cells (including tumour cells), glucose transporter-1 by rabbit-anti-Glut-1 (0.8 µg/mL; Cell Marque) as blood–brain barrier (BBB) marker, rabbit-anti-GFAP (1 µg/mL, clone EP6724; Cell Marque) to localize reactive astrocytes, and mouse-anti-PDGFR-beta (1:800; R&D) for pericytes. Slides were covered with anti-fading medium (DAKO, S3023) with DAPI. The full immunofluorescence staining protocol is provided in the Supplementary Material. The resulting images were visually assessed by the neuropathologists using ZEISS ZEN Miscroscopy software (Carl Zeiss AG, Oberkochen, Germany).

### [^68^Ga]Ga-PSMA-11 uptake and PSMA expression in samples

[^68^Ga]Ga-PSMA-11 uptake on PET was compared between samples with- and without PSMA expression in the respective H&E morphologies: tumour cells, microvasculature, pre-existent brain tissue and necrosis. Correlations were performed between the PSMA expression scores and [^68^Ga]Ga-PSMA-11 uptake as measured by SUVmax values from each 1 cm^3^ spherical VOI on PET. A subgroup analysis was performed in samples with PSMA-positive tumour cells and correlated to [^68^Ga]Ga-PSMA-11 uptake on PET. The correlations were separately performed for HGG and BM because of their biological differences.

### Statistical analyses

Statistical analysis was performed with Statistical Package for the Social Sciences (SPSS) version 24.0.0.1. (IBM Corporation, New York, USA). Normality of the data was determined using Kolmogorov–Smirnov Z-test for continuous variables, and reported by mean and standard deviation (± SD) for normally distributed variables and by median and range for non-normally distributed variables, respectively. The inter-rater variability was assessed using Cohen’s kappa.

Comparison between subgroups were performed using the Mann Whitney U test and correlations between PSMA expression and [^68^Ga]Ga-PSMA-11 uptake were assessed using the Spearman Rho correlation, as variables were non-normally distributed. A *P*-value < 0.05 was considered statistically significant.

## Results

### Patients and study procedures

Twelve patients with de novo or progressive HGG, including glioblastoma (IDH-wildtype) (*n* = 6) and oligodendroglioma (IDH-mutated, 1p/19q codeleted) (*n* = 1), or a secondary brain tumour from lung- (*n* = 4) or breast- (*n* = 1) carcinoma, were included. Diagnosis was confirmed by post-operative histopathology in all patients and did not differ from the initial diagnosis. Patient and tumour characteristics are displayed in Table 1.

**Table 1:** Patient and tumour characteristics

Patient no	Age	Sex	Diagnosis (grade, molecular subtype)	De novo or progression	Previous brain tumour therapy	Tumour size in cm^3a^	Tumour SUVmax at scan 3	Blood pool SUVmean at scan 1	TI PET-MRI to surgery, days	Samples, n	SUVmax at scan 3 (in each sample, separated by |)	PSMA vessel score^b^ (0–4)	PSMA other score^b^ (0–4)
High-grade glioma													
1	75	M	Glioblastoma (grade 4, IDHwt)	De novo	None	22.9	16.2	0.9	2	3	6.5 | 9.7 | 10.4	4 | 4 | 4	0 | 3 | 2
2	73	M	Glioblastoma (grade 4, IDHwt)	Progression	S, RTx, TMZ	22.2	5.7	1.0	17	4	2.5 | 5.7 | 1.0 | 0.5	0 | 2 | 0 | 0	3 | 3 | 3 | 4
3	41	M	Glioblastoma (grade 4, IDHwt)	Progression	S, RTx, TMZ,	0.2	19.8	0.7	6	3	2.0 | 6.7 | 10.0	1 | 2 | 0	3 | 3 | 2
4	61	M	Glioblastoma (grade 4, IDHwt)	De novo	None	17.1	8.2	0.8	9	2	6.6 | 7.1	4 | 4	4 | 3
5	69	M	Glioblastoma (grade 4, IDHwt)	Progression	S, RTx, TMZ	13.6	8.3	0.8	7	3	2.4 | 7.4 | 6.2	3 | 0 | 3	4 | 4 | 3
6	62	F	Glioblastoma (grade 4, IDHwt)	Progression	S, RTx, TMZ	24.7	4.7	0.8	14	2	3.8 | 4.6	4 | 3	3 | 2
7	72	M	Oligodendroglioma (grade 3, 1p/19q codel)	Progression	S, RTx, PCV	10.6	11.8	1.2	6	6	12.7 | 3.9 | 8.0 | 1.6 | 1.4 | 0.3	2 | 0 | 1 | 0 | 0 | 0	3 | 4 | 3 | 4 | 4 | 4
Brain metastasis													
8	70	M	Brain metastasis from lung cancer	Progression	S, RTx	14.8	12.4	0.8	6	9	NA | NA | 12.2 | NA | 9.3 | NA | NA | NA | 2.9	0 | 1 | 4 | 3 | 4 | 4 | 4 | 4 | 0	4 | 4 | 2 | 0 | 3 | 2 | 2 | 1 | 4
9	57	M	Brain metastasis from lung cancer	Progression	S, lorlatinib, RTx	10.6	11.7	1.0	3	1	5.2	1	4
10	63	F	Brain metastasis from breast cancer	De novo	None	30.0	10.6	0.8	7	3	10.6 | 5.8 | 6.4	0 | 4 | 4	2 | 1 | 1
11	63	M	Brain metastasis from lung cancer	Progression	RTx	13.7	10.3	1.2	6	4	5.3 | 10.3 | 5.3 | 7.2	3 | 0 | 0 | 2	2 | 3 | 4 | 4
12	59	M	Brain metastasis from lung cancer	Progression	RTx, C-P-P, sotorasib	5.6	5.0	1.1	6	3	4.2 | 4.3 | 4.3	0 | 1 | 0	4 | 4 | 4

^a^ Tumour size as calculated from contrast-enhancing area on MRI. ^b^ Scale defined as: 0 = none, 1 = limited, 2 = moderate, 3 = strong, 4 = very strong. Abbreviations: 1p/19q codel = 1p/19q chromosome codeletion, *C-P-P* Combined immunotherapy of carboplatin-pemetrexed-pembrolizumab, *F* female, *M* male, *n.a.* not applicable, *PET* positron-emission tomography, *PCV* procarbazine, lomustine and vincristine, *RTx* radiotherapy, *S* surgery, *TI* time interval, *TMZ* temozolomide

**Fig. 1:**
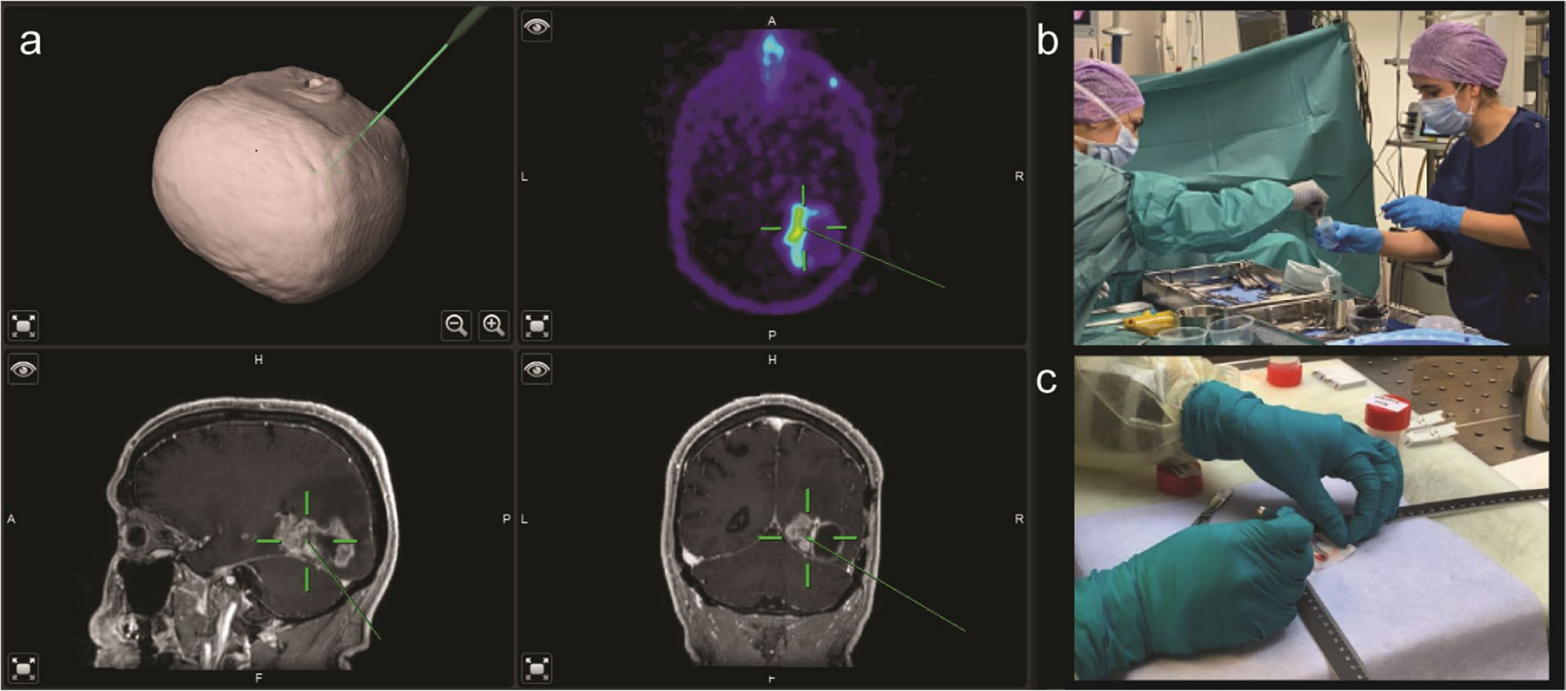
Illustrative case of PET-MR-guided per-operative tissue biopsy sampling. Representative example of patient no. 5 with progressive glioblastoma in the right parieto-occipital region who underwent re-resection during which the neurosurgeon used Brainlab © to per-operatively navigate to the tumour. The PET-MR images were loaded into the system (**a**) for biopsy sampling (**b**) of areas with low and high [^68^Ga]Ga-PSMA-11 uptake on PET. Preparation and fixation of the tissue was performed (**c**) for subsequent immunohistochemistry analysis. Abbreviations: MR = magnetic resonance, PET = positron emission tomography, PSMA = prostate-specific membrane antigen

All patients had a positive [^68^Ga]Ga-PSMA-11 PET scan with tumour uptake above blood pool activity levels. Uptake was heterogeneously distributed within tumours and between individuals. The median time interval between PET-MRI and biopsy sampling during surgery was 6 days (range: 2–17). A median of 3 samples were obtained per patient (range 1–9, total 43). Per-operative neuronavigation screenshots were available for post-procedural analysis in *n* = 37/43 tumour samples (*n* = 23 HGG, *n* = 14 BM) (Fig. 1). Six screenshots were not captured due to per-operative technical issues in the neuronavigation system. All biopsy sites corresponded to contrast-enhancing regions on MRI, consistent with previous observations that [^68^Ga]Ga-PSMA-11 uptake colocalizes with enhancing tumour tissue [10, 11].

### Immunohistochemistry analysis

H&E analysis showed that the majority of samples exhibited a composition of tumour cells, microvessels, pre-existent brain tissue and necrosis. Twenty-two out of 43 samples contained 50% or more tumour cells. Microvessels were detected in all samples. Four samples (1 out of 4 from HGG patient no. 7 and 3 out of 4 from BM patient no. 8) did not contain any detectable tumour cells and were accordingly described on H&E as pre-existent brain tissue (with undetectable tumour cell load) in the HGG sample, and as brain tissue with reactive changes in the BM samples. In two samples (patient no. 3 and no. 1) less than 10% of the tissue were tumour cells. Two samples (both from BM patient no. 12) were predominantly hemorrhagic, due to difficulty during the procedure, limiting accurate evaluation of tumour cells and microvessels. Given the considerable [^68^Ga]Ga-PSMA-11 uptake observed preoperatively (SUVmax > 4), we attribute this to a sampling error. The two control samples constituted for 100% of healthy brain tissue with only some reactive, possible post-mortem, changes. All samples were included in the analysis to account for tumour microenvironment heterogeneity.

The inter-rater agreement for scoring of PSMA expression was considered “very good” (k = 0.81, *P* < 0.001) for microvasculature (median score: 2, range: 0–4), and “moderate” (k = 0.44, *P* < 0.01) for non-vascular structures (median: 3, range: 0–4).

### [^68^Ga]Ga-PSMA-11 uptake and PSMA expression in samples

**Fig. 2:**
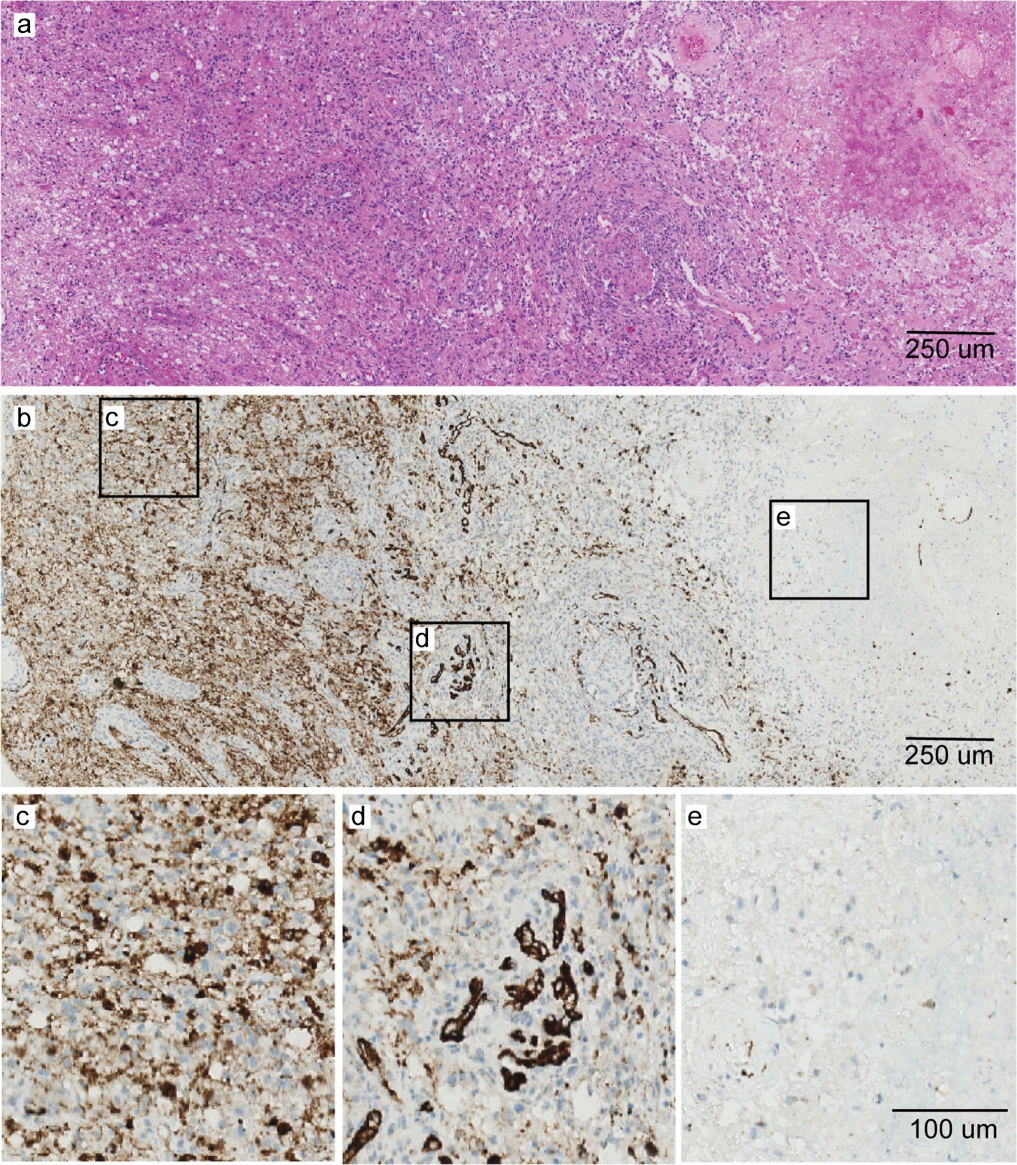
Representative illustration of PSMA expression pattern in a HGG sample. Overview snapshot of patient no. 1 (**a**, H&E) showing three distinct morphologies in the tumour biopsy; including a tumour cell dense area, tumour microvasculature around tumour cells and necrotic tissue with corresponding PSMA expression (**b**) in respectively cytoplasm and some tumour cells (**c**), in tumour microvasculature (**d**) and absent PSMA expression in necrotic tissue (**e**). Abbreviations: HGG = high-grade glioma, PSMA = prostate-specific membrane antigen

**Fig. 3:**
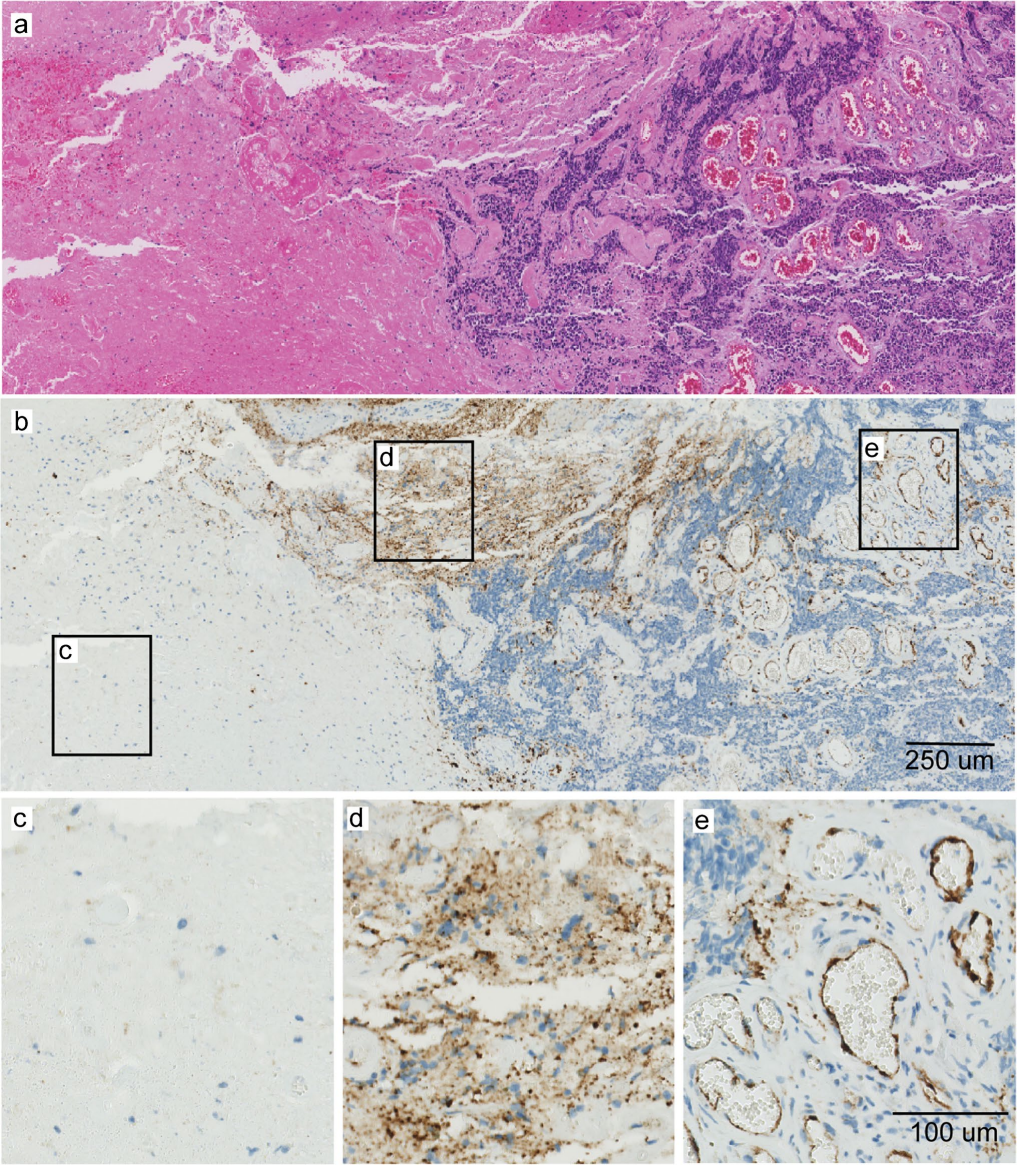
Representative illustration of PSMA expression pattern in a BM sample. Overview snapshot of patient no. 8 (**a**, H&E) showing three distinct morphologies in the tumour biopsy including necrotic tissue, some pre-existent tissue and tumour cell dense area with tumour microvasculature. PSMA expression (**b**) was absent in necrotic tissue (**c**), weak/aspecific in pre-existent cytoplasm (**d**) and absent on tumour cells, but strong in tumour microvessels (**e**). Abbreviations: BM = brain metastasis, PSMA = prostate-specific membrane antigen

Representative examples showing the regional pattern of PSMA expression in tumour are visualised in Fig. 2 (HGG) and Fig. 3 (BM).

PSMA expression was found on *tumour cells*, in only a subset of samples: 10 out of 23 (43%, of which strong in 8 out of 10) HGG and 3 out of 20 (15%, of which strong in none) BM, independent of tumour cell density. At the location of the HGG samples with PSMA-positive tumour cells (*n* = 10), [^68^Ga]Ga-PSMA-11 uptake did not differ significantly compared to areas from samples without PSMA-positive tumour cells (*n* = 12) (SUVmax, median, range: 6.9, 0.3–10.4 versus 4.7, 0.5–12.7; *P* = 0.26). In the BM group, due to the negligible PSMA expression on tumour cells, no comparison was made with PET uptake. In the *n* = 2/4 samples with available screenshots (*n* = 1 HGG, *n* = 1 BM) where no detectable tumour cells were present, SUVmax values were low (1.6 and 2.9).

PSMA expression was detected on endothelial cells of *microvessels* in the majority of samples: 14 out of 23 (61%, of which strong in 9 out of 14) HGG and 13 out of 20 (65%, of which strong in 9 out of 13) BM samples, respectively, which was dependent on tumour cell density. PSMA-positive microvessels were localised in tumour cell dense regions and not found in pre-existent brain tissue regions apart from 4 samples, but only limited in some small vessels. At the location of HGG samples with PSMA expression on microvessels, [^68^Ga]Ga-PSMA-11 uptake did differ significantly compared to areas from samples without PSMA expression on microvessels (SUVmax median, range: 6.6, 2.0–12.7 versus 1.6, 0.3–10.0, *P* < 0.05). At the location of BM samples with PSMA-positive microvessels, [^68^Ga]Ga-PSMA-11 uptake was higher compared to areas from samples without PSMA-positive microvessels, but did not reach statistical significance (SUVmax median, range: 6.1, 4.3–12.2 versus 4.8, 2.9–10.6, *P* = 0.52).

PSMA expression was also detected on individual (reactive) glial cells, neuropil and fibroblasts in the tumour samples. Both *pre-existent brain* and tumour cell dense areas in tumour samples showed PSMA-positivity in these cells: 22 out of 23 HGG and 19 out of 20 BM samples. Due to the homogenous PSMA expression in cells other than tumour cells or microvessels, across both HGG and BM tumour samples, no comparison was made with PET uptake. Only negligible PSMA expression was detected in the *n* = 2 *healthy control* samples in these cells and in microvasculature: limited fibrillary staining in glial cells (IHC score 1) was detected and moderate PSMA expression (IHC score 2) in some endothelial cells of normal vessels.

PSMA expression was not detected in *necrosis*, neither in HGG nor BM samples.

### Multiplex immunofluorescence staining of tumour-microenvironment in HGG

**Fig. 4:**
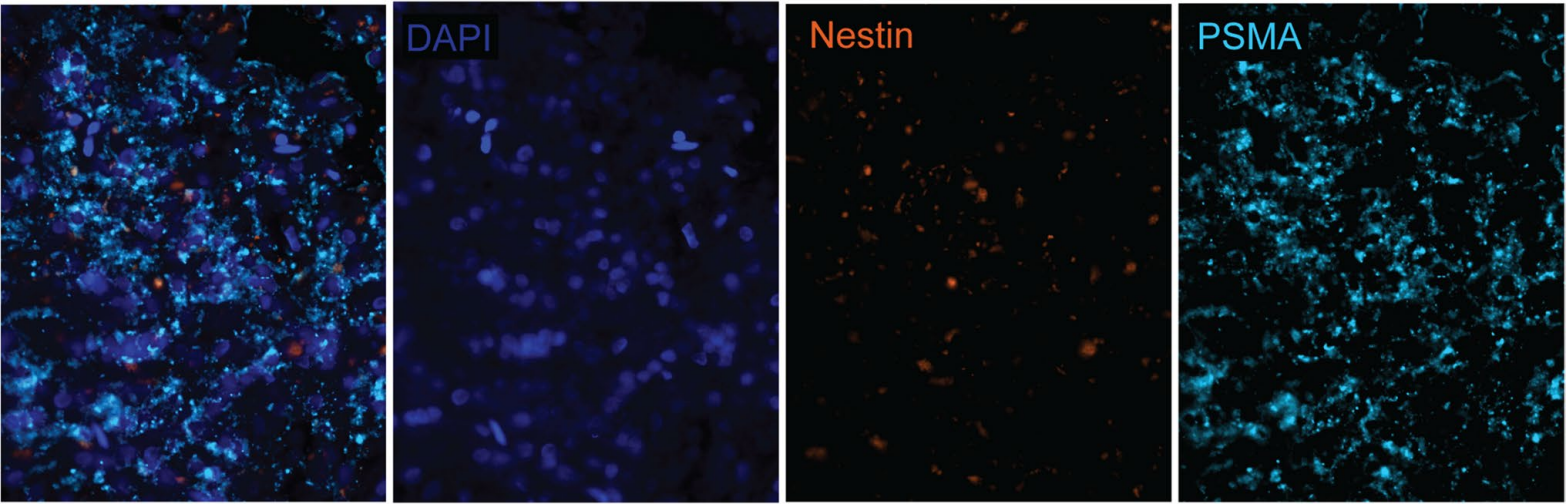
Multiplex immunofluorescence showing PSMA and nestin co-expression in a subset of tumour cells. Snapshot figures of representative tumour tissue slice (patient no. 5) showing from left to right a fused multiplex, expression of DAPI (cell nuclei), nestin and PSMA. High PSMA expression was found in this population of tumour cells but only a subset also showed nestin expression. Abbreviations: DAPI = 4′,6-diamidino-2-phenylindole, PSMA = prostate-specific membrane antigen

**Fig. 5:**
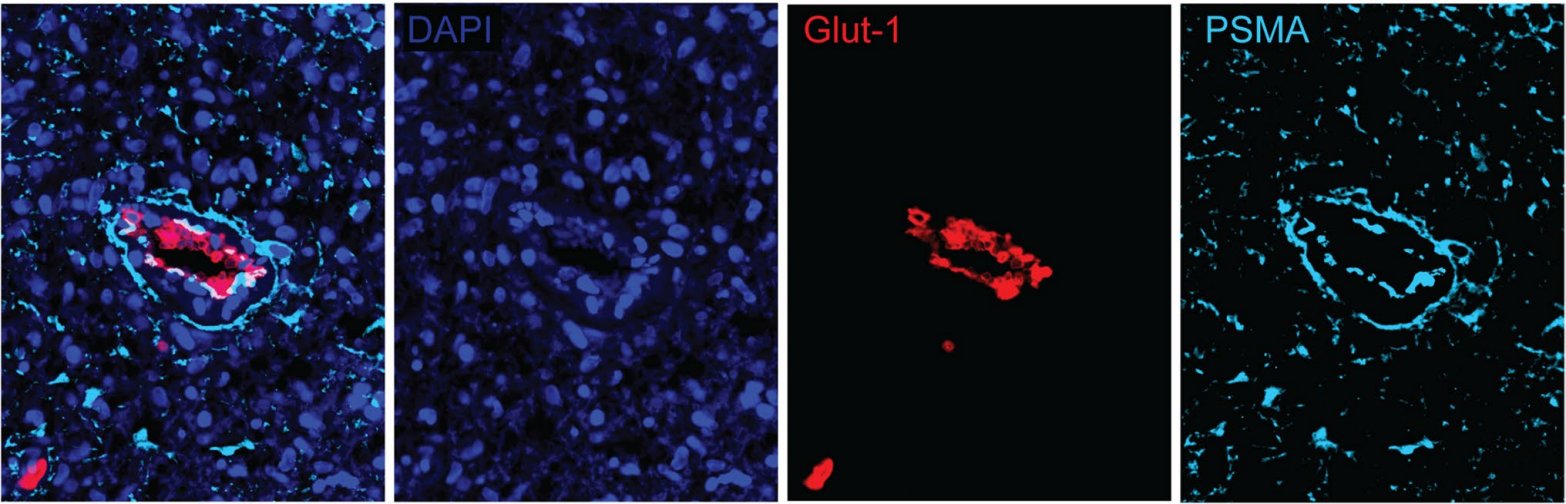
Multiplex immunofluorescence showing PSMA expression in tumour microvasculature. Snapshot figures of representative tumour tissue slice (patient no. 4) showing from left to right a fused multiplex, expression of DAPI (cell nuclei), Glut-1 and PSMA. PSMA expression was found on endothelial cells of vessels in tumour cell dense areas. Abbreviations: DAPI = 4′,6-diamidino-2-phenylindole, Glut-1 = glucose transporter-1, PSMA = prostate-specific membrane antigen

**Fig. 6:**
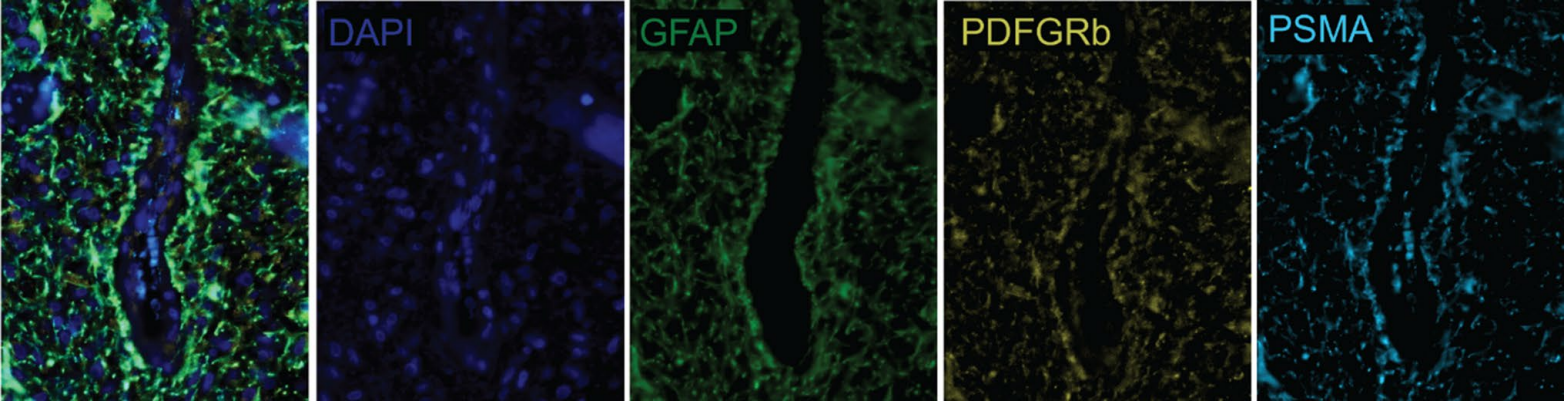
Multiplex immunofluorescence showing co-expression of GFAP, PDFGRb and PSMA. Snapshot figures of representative tumour tissue slice (patient no. 4) showing from left to right a fused multiplex, expression of DAPI (cell nuclei), GFAP, PDGFRb and PSMA around a microvessel in a tumour cell dense area. PSMA expression colocalised with GFAP, which showed extensive expression, highlighting the astrocyte endfeets connected to the vessel. PSMA expression also colocalized in the endfeets of pericytes. The aspecific staining (in cyanin blue) in the lumen of the vessels are blood cells and not PSMA. Abbreviations: DAPI = 4′,6-diamidino-2-phenylindole, GFAP = glial fibrillary acidic protein, PDGFRb = platelet-derived growth factor receptor beta, PSMA = prostate-specific membrane antigen

In the selected HGG samples (*n* = 3), multiplex immunofluorescence staining confirmed only limited colocalization of PSMA with the marker nestin, i.e., with neuroepithelial stem cells including tumour cells, in line with the immunohistochemistry results (Fig. 4). Besides, nestin expression was only found in a subset of the tumour cells that were identified on H&E. Some endothelial cells of microvessels (identified by colocalization with Glut-1) in tumour cell dense regions also showed nestin expression. Colocalization of PSMA with Glut-1 was confirmed specifically on the endo-luminal side of vessels (Fig. 5). Overall, at the borders of tumour cell dense to pre-existent tissue (defined as peritumoural area), PSMA expression colocalised with astrocyte endfeet, indicated from GFAP co-expression, and with perictyes, indicated from PDFGRb co-expression (Fig. 6) – these PSMA-positive astrocytes and pericytes were lining the microvessels.

### Relation between [^68^Ga]Ga-PSMA-11 uptake intensity and PSMA expression

**Fig. 7:**
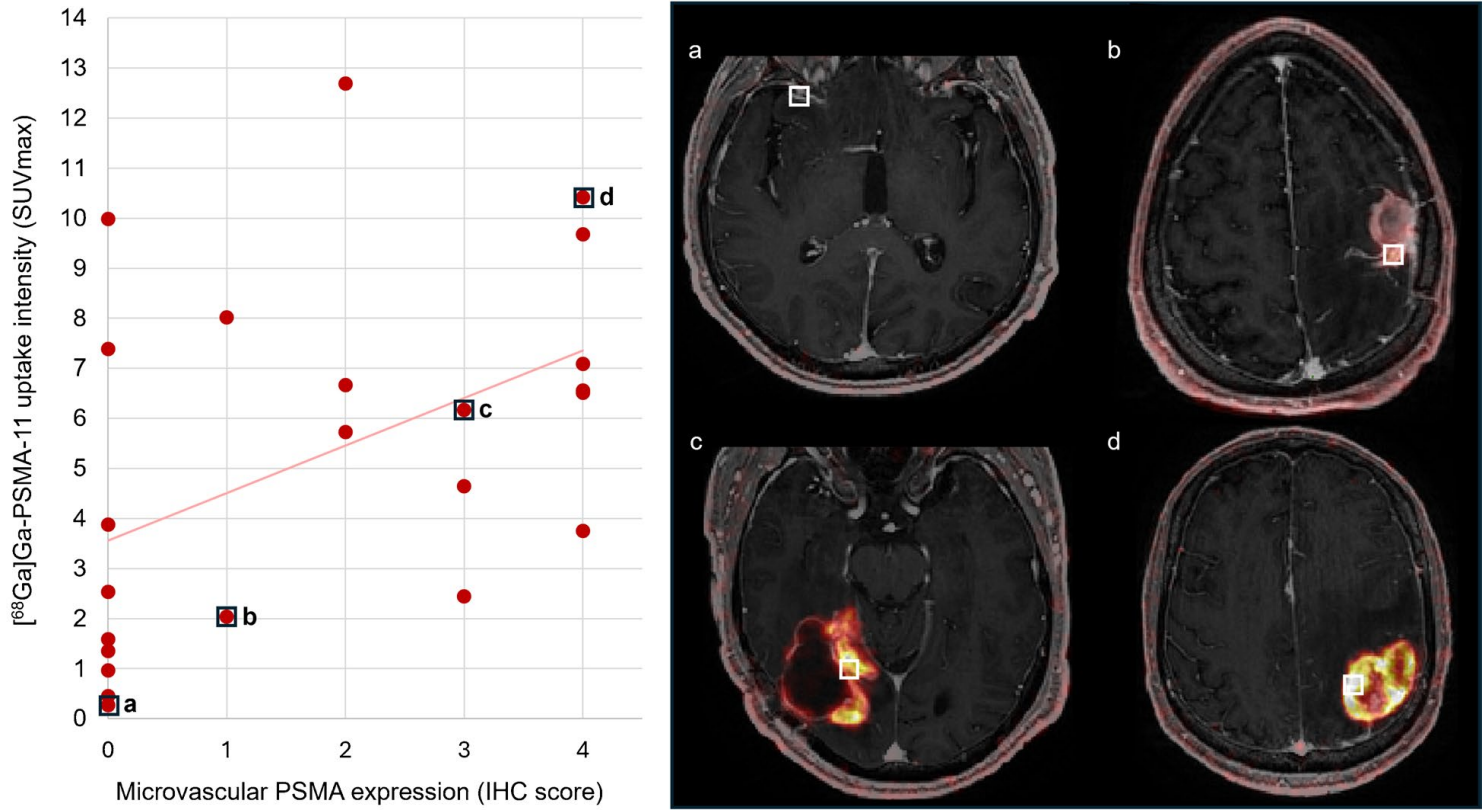
Scatterplot of the correlation between [^68^Ga]Ga-PSMA-11 uptake intensity on PET (SUVmax) and PSMA expression in tumour microvessels (IHC score) in HGG samples (r = 0.487, *P* < 0.01); highlighting four representative fused PET with post-contrast T1w MR images at the respective biopsy location. The biopsy location is indicated with a white circle (patient no. 7 (**a**), 3 (**b**), 5 (**c**), 1 (**d**)). Abbreviations: BM = brain metastasis, HGG = high-grade glioma, IHC = immunohistochemistry, PET = positron-emission tomography, PSMA = prostate-specific membrane antigen

A significant correlation was found between [^68^Ga]Ga-PSMA-11 uptake intensity on PET measured by SUVmax (r = 0.487, *P* < 0.01) and PSMA expression levels in tumour microvasculature in HGG samples, but not in BM samples (P > 0.1) (Fig. 7). The subgroup analysis of samples with PSMA-positive tumour cells did not show a correlation between [^68^Ga]Ga-PSMA-11 uptake intensity and PSMA expression levels in HGG (P > 0.05).

## Discussion

In this prospective clinical imaging and multiregional tumour biopsy study of patients with malignant brain tumours, the direct correlation between [^68^Ga]Ga-PSMA-11 uptake on PET-MRI and PSMA expression in tumour tissue was assessed. This allowed for in-depth structural analysis of PSMA expression and assessment of intra-tumoural, intra-lesional and inter-individual heterogeneity. We observed strong and extensive PSMA expression in microvessels correlating to tumour density, in both HGG (61%) and BM (65%) while vessels outside tumour cell dense regions did not show any PSMA expression, corresponding with our findings in the pre-existent brain controls. A statistically significant correlation was found between [^68^Ga]Ga-PSMA-11 uptake intensity and PSMA expression in the tumour microvessels in HGG samples. Interestingly, 35% of HGG samples showed strong PSMA expression on individual tumour cells; however, no correlation with [^68^Ga]Ga-PSMA-11 uptake intensity was found. In BM, PSMA expression on individual tumour cells was identified in only 15% of samples, none of which demonstrated strong expression.

In-depth knowledge of the precise localization of target expression is essential for selecting the most appropriate radionuclide for future RLT strategies. Our findings indicate that [^68^Ga]Ga-PSMA-11 uptake in malignant brain tumours predominantly reflects binding to the tumour microvasculature. This suggests that alpha-emitting RLT may be less effective in directly targeting tumour DNA due to the limited path length of alpha particles (~ 50–100 µm). In contrast, beta-emitting radionuclides, such as lutetium-177, offer greater potential owing to their longer tissue penetration range (up to several millimeters), which may allow for both damage to the tumour-associated vasculature and irradiation of adjacent tumour cells. Nevertheless, as [^68^Ga]Ga-PSMA-11 consistently co-localizes with contrast-enhancing tumour regions – presumed to reflect areas of blood–brain barrier (BBB) disruption [11], the overall efficacy of PSMA-based RLT may be limited by incomplete irradiation of infiltrating tumour cells located beyond the contrast-enhancing margins. In the present study, we did not distinguish between de novo (*n* = 3) and progressive (*n* = 9) disease, nor did we stratify by tumour grade, as our primary aim was to evaluate PSMA a potential target for RLT in patients with otherwise limited or no remaining treatment option, regardless of prior therapy or disease setting.

Only two studies have to date investigated the direct correlation between radioligand uptake on PET and PSMA expression in corresponding tumour samples [9, 10]. The study by Truckenmueller et al. reported slightly lower SUVmax values in tumour (median, range 4.5, 3.7–6.2 versus 5.9, 0.4–13.0) and found a trend between [^68^Ga]Ga-PSMA uptake intensity and PSMA expression on tumour vessels, measured in eleven HGG patients. In their study, however, samples were taken before and not after PET imaging, like in our study, with a long time interval between surgery and PET imaging (median 12 months, range 9–14, versus 6 days, range 2–17). Besides, only a single biopsy was obtained per patient. As such, heterogeneity in expression patterns within the tumour micro-environment in relation to radioligand uptake had not yet been accurately established. The other study was performed by our own group in a multicentre setting [10], in which strong PSMA expression in tumour microvessels was found but without a correlation with uptake intensity on PSMA PET, most likely explained by tumour heterogeneity and sampling errors and/or differences between the PSMA-radioligands used (between centres).

This study is one of the few to date that offers a direct and in-depth method for comparisons, addressing a gap largely attributed to the lack of consensus on the most optimal and reliable approach for assessing target expression. Since 1999, several approaches have been reported in literature with regard to scoring and quantification of PSMA expression, resulting in highly variable outcomes. For example, five studies reported that PSMA is expressed in HGG tumour cells and individual cells not identifiyable as tumour cells (but no endothelial cells) in less than 2% to up to 50% of samples [5, 6, 14, 15]. There is an increasing need for standardized, potentially semi-automated systems for staining and scoring to evaluate and compare image-guided target expression through immunohistochemistry. This requires particular attention to the standardization of antibody usage, staining protocols, and their application to large, comprehensive validation datasets, to enhance and optimize reproducibility. In this study we used the most advanced method currently available –images from multiregional targeted biopsies taken from areas with low and high [^68^Ga]Ga-PSMA-11 uptake, which were assessed by two independent evaluators using a scale-based scoring system.

This study had some limitations. First, some of the samples could not be accurately assessed as they consisted of mostly blood and necrotic tissue in 2/5 BM patients. This was explained by the neurosurgeon as due to sampling difficulties during surgery. However, since we obtained multiregional image-guided samples per patient we were still able to assess target expression patterns in BM. The small sample and hence lack of power may however explain that we could not confirm a correlation between [^68^Ga]Ga-PSMA-11 uptake and PSMA-positive microvessels in BM. This could also be attributed to a true underlying biological and morphological difference: while microvascular neovascularisation is a prominent histological feature of HGG [16], it is a less distinctive feature in BM. Second, the interpretation of the co-expression of PSMA with the tumour-cell marker nestin was hampered by the fact that nestin itself was not expressed in all tumour cells. This may be explained by differential losing or gaining of nestin expression, as literature shows that subpopulations of glioma cells can differ in their mutational status [17–20]. In line with a previous study, we also found nestin expression on subpopulations of endothelial cells [21]. To our knowledge, no markers yet exist that are specific for only brain tumour cells of the IDH-wildtype (off note: accurate stainings for IDH1 R132H mutant tumour cells do exist); showing the need to develop better high-grade glioma cell-markers. Finally, a well-known methodological issue is the mismatch and possible discrepancy between PET(-MRI) and immunohistochemistry. For example, brain shift during open surgery tissue sampling and differences in resolution between the two techniques (i.e., 3 mm slices on PET and 4 µm slices in tissue) can influence the accuracy of the measurements. We therefore aimed to obtain samples centrally from distinct (i.e., distant) regions of low and high [^68^Ga]Ga-PSMA-11 uptake as to limit location errors as much as possible. Strategies to further improve precision are for example to homogeneously obtain similar-sized cylindrical samples, as suggested in literature [13], while recording the coordinates before dural opening, demanding close collaborations between nuclear radiologists and neurosurgeons with in-house expertise in the technique.

In conclusion, our findings demonstrate that future PSMA-based RLT strategies hold promise for treatment of malignant brain tumours. The microvascular expression pattern is crucial information, as it indicates that alpha-based therapy will likely not reach the tumour DNA sufficiently. Future clinical bed-to-bench-and-back studies need to establish these radiobiological effects of PSMA-based RLT in the tumour micro-environment.

## Supplementary Information

Below is the link to the electronic supplementary material.

Supplementary file1 (DOCX 21 KB)

## Data Availability

The datasets generated during and/or analysed during the current study are available from the corresponding author on reasonable request.

## References

[1] Sgouros G, Bodei L, McDevitt MR, Nedrow JR. Radiopharmaceutical therapy in cancer: clinical advances and challenges. Nat Rev Drug Discov. 2020;19:589–608. 32728208 10.1038/s41573-020-0073-9 PMC7390460

[2] Sartor O, de Bono J, Chi KN, Fizazi K, Herrmann K, Rahbar K, et al. Lutetium-177-PSMA-617 for metastatic castration-resistant prostate cancer. N Engl J Med. 2021;385:1091–103. 34161051 10.1056/NEJMoa2107322 PMC8446332

[3] Stopa BM, Crowley J, Juhász C, Rogers CM, Witcher MR, Kiser JW. Prostate-specific membrane antigen as target for neuroimaging of central nervous system tumors. Mol Imaging. 2022;2022:5358545. 35517711 10.1155/2022/5358545 PMC9042374

[4] McBriar JD, Shafiian N, Scharf S, Boockvar JA, Wernicke AG. Prostate-specific membrane antigen use in glioma management: past, present, and future. Clin Nucl Med. 2024;49:806–16. 38968568 10.1097/RLU.0000000000005365

[5] Traub-Weidinger T, Poetsch N, Woehrer A, Klebermass EM, Bachnik T, Preusser M, et al. PSMA Expression in 122 Treatment Naive Glioma Patients Related to Tumor Metabolism in (11)C-Methionine PET and Survival. J Pers Med. 2021;11:624. 34209106 10.3390/jpm11070624 PMC8305688

[6] Holzgreve A, Biczok A, Ruf VC, Liesche-Starnecker F, Steiger K, Kirchner MA, et al. PSMA expression in glioblastoma as a basis for theranostic approaches: a retrospective, correlational panel study including immunohistochemistry, clinical parameters and PET imaging. Front Oncol. 2021;11:646387. 33859946 10.3389/fonc.2021.646387 PMC8042319

[7] Bertagna F, Albano D, Cerudelli E, Gazzilli M, Giubbini R, Treglia G. Potential of radiolabeled PSMA PET/CT or PET/MRI diagnostic procedures in gliomas/glioblastomas. Curr Radiopharm. 2020;13:94–8. 31625482 10.2174/1874471012666191017093721 PMC7527542

[8] Lee ST, Burvenich I, Scott AM. Novel target selection for nuclear medicine studies. Semin Nucl Med. 2019;49:357–68. 31470931 10.1053/j.semnuclmed.2019.06.004

[9] Truckenmueller P, Graef J, Scheel M, Vajkoczy P, Capper D, Kaul D, et al. [(68)Ga]Ga-PSMA PET/MRI, histological PSMA expression and preliminary experience with [(177)Lu]Lu-PSMA therapy in relapsing high-grade glioma. Front Oncol. 2022;12:980058. 36119502 10.3389/fonc.2022.980058 PMC9478729

[10] van Lith SAM, Pruis IJ, Tolboom N, Snijders TJ, Henssen D, Ter Laan M, et al. PET imaging and protein expression of prostate-specific membrane antigen in glioblastoma: a multicenter inventory study. J Nucl Med. 2023;64:1526–31. 37652540 10.2967/jnumed.123.265738

[11] Pruis IJ, van Doormaal PJ, Balvers RK, van den Bent MJ, Harteveld AA, de Jong LC, et al. Potential of PSMA-targeting radioligand therapy for malignant primary and secondary brain tumours using super-selective intra-arterial administration: a single centre, open label, non-randomised prospective imaging study. EBioMedicine. 2024;102:105068. 38518652 10.1016/j.ebiom.2024.105068 PMC10981001

[12] Verburg N, Koopman T, Yaqub M, Hoekstra OS, Lammertsma AA, Schwarte LA, et al. Direct comparison of [(11)C] choline and [(18)F] FET PET to detect glioma infiltration: a diagnostic accuracy study in eight patients. EJNMMI Res. 2019;9:57. 31254208 10.1186/s13550-019-0523-8 PMC6598977

[13] Verburg N, Koopman T, Yaqub MM, Hoekstra OS, Lammertsma AA, Barkhof F, et al. Improved detection of diffuse glioma infiltration with imaging combinations: a diagnostic accuracy study. Neuro Oncol. 2020;22:412–22. 31550353 10.1093/neuonc/noz180 PMC7058442

[14] Tanjore Ramanathan J, Lehtipuro S, Sihto H, Tóvári J, Reiniger L, Téglási V, et al. Prostate-specific membrane antigen expression in the vasculature of primary lung carcinomas associates with faster metastatic dissemination to the brain. J Cell Mol Med. 2020;24:6916–27. 32390293 10.1111/jcmm.15350 PMC7299712

[15] Vallejo-Armenta P, Soto-Andonaegui J, Villanueva-Pérez RM, González-Díaz JI, Contreras-Contreras K, Bautista-Wong CG, et al. [(99m)Tc]Tc-iPSMA SPECT brain imaging as a potential specific diagnosis of metastatic brain tumors and high-grade gliomas. Nucl Med Biol. 2021;96–97:1–8. 33640681 10.1016/j.nucmedbio.2021.02.003

[16] Louis DN, Perry A, Wesseling P, Brat DJ, Cree IA, Figarella-Branger D, et al. The 2021 WHO classification of tumors of the central nervous system: a summary. Neuro Oncol. 2021;23:1231–51. 34185076 10.1093/neuonc/noab106 PMC8328013

[17] Li M, Li G, Kiyokawa J, Tirmizi Z, Richardson LG, Ning J, et al. Characterization and oncolytic virus targeting of FAP-expressing tumor-associated pericytes in glioblastoma. Acta Neuropathol Commun. 2020;8:1–13. 33308315 10.1186/s40478-020-01096-0 PMC7730751

[18] Ma YH, Mentlein R, Knerlich F, Kruse ML, Mehdorn HM, Held-Feindt J. Expression of stem cell markers in human astrocytomas of different WHO grades. J Neurooncol. 2008;86:31–45. 17611714 10.1007/s11060-007-9439-7

[19] Chinnaiyan P, Wang M, Rojiani AM, Tofilon PJ, Chakravarti A, Ang KK, et al. The prognostic value of nestin expression in newly diagnosed glioblastoma: report from the Radiation Therapy Oncology Group. Radiat Oncol. 2008;3:32. 18817556 10.1186/1748-717X-3-32 PMC2563009

[20] Schiffer D, Manazza A, Tamagno I. Nestin expression in neuroepithelial tumors. Neurosci Lett. 2006;400:80–5. 16529857 10.1016/j.neulet.2006.02.034

[21] Maderna E, Salmaggi A, Calatozzolo C, Limido L, Pollo B. Nestin, PDGFRbeta, CXCL12 and VEGF in glioma patients: different profiles of (pro-angiogenic) molecule expression are related with tumor grade and may provide prognostic information. Cancer Biol Ther. 2007;6:1018–24. 17611402 10.4161/cbt.6.7.4362

