# Supplementary material for: PSMA radioligand uptake correlates with PSMA expression in high-grade glioma and brain metastasis: insights from a prospective PET-MRI guided multiregional biopsy study
Source: Eur J Nucl Med Mol Imaging. 2025 May 26;52(13):4870–81. doi: 10.1007/s00259-025-07338-4 (PMC12589371; doi:10.1007/s00259-025-07338-4)
Supplement: Supplementary file 1 — Supplementary file1 (DOCX 21 KB) [file 259_2025_7338_MOESM1_ESM.docx]

**Supplementary Material**

[*PET-MRI acquisition details* 2](#_Toc190427121)

[*Multiplex immunofluorescence staining protocol* 3](#_Toc190427122)

# *PET-MRI acquisition details*

[^68^Ga]Ga-PSMA-11 was prepared in-house using the commercially purchased precursor PSMA-11 (ABX, Radeberg, Germany). Scans were acquired on a 3.0T whole-body hybrid PET-MR system (Signa PET-MR, GE Healthcare, USA)) at 90 minutes (min; 1.5 hrs), 165 min (2.75 hrs) and 240 min (4 hrs) after intravenous injection (post-injection, p.i.) of 1.5 MBq/kg [^68^Ga]Ga-PSMA-11. At each time-point a single bed position PET scan of the brain was acquired for 15 min (90 min and 165 min p.i.) or 20 min (240 min p.i., 10 min pre- and post-gadolinium administration) in ‘list mode’ to enable dynamic (i.e., continuous) acquisition. At each timepoint, a single bed position (bp) PET scan of the brain was acquired for 15 min. The first scan was followed by a whole-body scan (from skull to vertex to thighs) of 5 bp (each 3 min). Standard 4-tissue (air, lung, water, fat) MR DIXON based attenuation maps (MRAC) were acquired simultaneously; this was combined with a standard MR ZTE scan for the head, that adds bone tissue to the MRAC. In addition, MR sequences were acquired simultaneously during each PET bed position for anatomical localization. PET list-mode data were binned in one frame of 15 (90 and 165 min p.i.) and 20 (240 min p.i.) minutes for reconstruction of static PET images (with a slice thickness of 2.7 mm). All PET images were reconstructed using a block sequential regularized expectation maximization (BSREM) algorithm using time-of-flight (ToF) information, point-spread-functions (PSF) and a beta value of 300. Corrections for attenuation, scatter and random coincidences were applied.

# *Multiplex immunofluorescence staining protocol*

Following deparaffinization and heat-induced antigen retrieval with CC1 (#950-500, Ventana) for 40 minutes at 97°C, multiplex immunofluorescence (IF) stainings were performed on the Ventana Benchmark Discovery (Ventana Medical Systems Inc.). Antibody information can be found in the Supplementary Table 1 below.

For the first panel the tissue samples were incubated firstly with PSMA for 36 minutes at 37˚C followed by detection with omnimap anti-rabbit HRP (#760-4311, Ventana) followed by visualization with DCC (#760-235, Ventana) for 4 minutes. Antibody denature step was performed using CC2 for 20 minutes at 100˚C. Second, Glut-1 was incubated for 24 minutes at 37 ˚C followed by detection with omnimap anti-rabbit HRP for 20 minutes followed by visualization with Red610 (#760-245, Ventana) for 8 minutes. Thirdly, Nestin was incubated for 32 minutes at 37˚C followed by detection with omnimap anti-mouse HRP (#760-4310, Ventana) followed by visualization with Cy5 (#760-238, Ventana) for 4 minutes. At lasted, GFAP was incubated for 24 minutes at 37 ˚C followed by detection with omnimap anti-rabbit HRP for 20 minutes at 37 ˚C followed by visualization with FAM (#760-243, Ventana) for 4 minutes.

For the seconded panel the tissue samples were incubated firstly with PDGFR-β for 32 minutes at 37˚C followed by an amplification step and by detection with omnimap anti-mouse HRP for 20 minutes followed by visualization with R6G for 4 minutes. Antibody denature step was performed using CC2 for 20 minutes at 100˚C. Secondly, PSMA was incubated for 36 minutes at 37˚C followed by detection with omnimap anti-rabbit HRP followed by visualization with DCC for 4 minutes. Antibody denature step was performed using CC2 for 20 minutes at 100˚C. Thirdly, Nestin was incubated for 32 minutes at 37˚C followed by detection with omnimap anti-mouse HRP followed by visualization with Cy5 for 4 minutes. At lasted, GFAP was incubated for 24 minutes at 37 ˚C followed by detection with omnimap anti-rabbit HRP for 20 minutes at 37 ˚C followed by visualization with FAM for 4 minutes. The slides were covered with anti-fading medium (DAKO, S3023) with DAPI.

The resulting images were visually assessed by the neuropathologists using ZEISS ZEN Microscopy Software (Carl Zeiss AG, Oberkochen, Germany).

**Supplementary Table 1. Antibody information**

| **Antibody** | **Type** | **Concentration/Dilution** | **Company** | **Clone** |
| --- | --- | --- | --- | --- |
| PSMA | Anti-rabbit | 13 µg/ml | Cell Marque | EP192 |
| Glut-1 | Anti-rabbit | 0,8 µg/ml | Cell Marque | - |
| Nestin | Anti-mouse | 1:25600 | NovusBio | - |
| GFAP | Anti-rabbit | 1 µg/ml | Cell Marque | EP6724 |
| PDGFRb | Anti-mouse | 1:800 | R&D | - |

Abbreviations: Glut-1 = glucose transporter-1, GFAP = glial fibrillary acidic protein, PDGFRb = Platelet-derived growth factor receptors beta, PSMA = prostate-specific membrane antigen.
